# Chronic administration of R-flurbiprofen attenuates learning impairments in transgenic amyloid precursor protein mice

**DOI:** 10.1186/1471-2202-8-54

**Published:** 2007-07-24

**Authors:** Thomas Kukar, Sonya Prescott, Jason L Eriksen, Vallie Holloway, M Paul Murphy, Edward H Koo, Todd E Golde, Michelle M Nicolle

**Affiliations:** 1 Department of Neuroscience, Mayo Clinic College of Medicine, Jacksonville, FL 32224, USA; 2 Department of Molecular and Cellular Biochemistry, University of Kentucky, Lexington, KY 40536, USA; 3 Department of Neurosciences, University of California, San Diego, La Jolla, California, USA; 4 Departments Internal Medicine/Gerontology and the Department of Physiology and Pharmacology, Wake Forest University School of Medicine, Winston-Salem, NC 27157, USA

## Abstract

**Background:**

Long-term use of non-steroidal anti-inflammatory drugs (NSAIDs) is associated with a reduced incidence of Alzheimer's disease (AD). We and others have shown that certain NSAIDs reduce secretion of Aβ42 in cell culture and animal models, and that the effect of NSAIDs on Aβ42 is independent of the inhibition of cyclooxygenase by these compounds. Since Aβ42 is hypothesized to be the initiating pathologic molecule in AD, the ability of these compounds to lower Aβ42 selectively may be associated with their protective effect. We have previously identified *R*-flurbiprofen (tarenflurbil) as a selective Aβ42 lowering agent with greatly reduced cyclooxygenase activity that shows promise for testing this hypothesis. In this study we report the effect of chronic *R*-flurbiprofen treatment on cognition and Aβ loads in Tg2576 APP mice.

**Results:**

A four-month preventative treatment regimen with *R*-flurbiprofen (10 mg/kg/day) was administered to young Tg2576 mice prior to robust plaque or Aβ pathology. This treatment regimen improved spatial learning as assessed by the Morris water maze, indicated by an increased spatial bias during the third probe trial and an increased utilization of a place strategy to solve the water maze. These results are consistent with an improvement in hippocampal- and medial temporal lobe-dependent memory function. A modest, though not statistically significant, reduction in formic acid-soluble levels of Aβ was also observed. To determine if R-flurbiprofen could reverse cognitive deficits in Tg2576 mice where plaque pathology was already robust, a two-week therapeutic treatment was given to older Tg2576 mice with the same dose of *R*-flurbiprofen. This approach resulted in a significant decrease in Aβ plaque burden but no significant improvement in spatial learning.

**Conclusion:**

We have found that chronic administration of *R*-flurbiprofen is able to attenuate spatial learning deficits if given prior to plaque deposition in Tg2576 mice. Given its ability to selectively target Aβ42 production and improve cognitive impairments in transgenic APP mice, as well as promising data from a phase 2 human clinical trial, future studies are needed to investigate the utility of *R*-flurbiprofen as an AD therapeutic and its possible mechanisms of action.

## Background

Alzheimer's disease (AD) is the most common form of dementia, and results in a progressive, irreversible decline in memory and cognitive function. One of the pathological hallmarks of the Alzheimer's brain is the presence of aggregated amyloid beta (Aβ) peptide in extracellular proteinaceous deposits in the parenchyma (senile plaques), and cerebral blood vessels [1]. Aβ species with different amino- and carboxyl-termini are constitutively produced from the amyloid β precursor protein (APP) through sequential proteolysis by β- and γ-secretases [2]. In most cases, a 40 amino acid form of Aβ (Aβ40) is the major secreted product of these cleavages. The 42 amino acid form of Aβ (Aβ42), although secreted at much lower levels than Aβ40, has been implicated as the initiating molecule in the pathogenesis of AD [3].

Aβ42 is more amyloidogenic than Aβ40, and is deposited earlier and more consistently than Aβ40 in the AD brain parenchyma. Significantly, mutations in presenilin 1 (PS1), presenilin 2 (PS2), and APP genes linked to early onset genetic forms of AD perturb Aβ peptide levels or in rare cases directly alter the Aβ sequence in a way that increase the propensity of the mutant Aβ to aggregate and form fibrils. The vast majority of these AD-linked mutations selectively increase the relative levels of Aβ42 peptides (reviewed in [4,5]). Small shifts in Aβ42 production have a tremendous impact on the development of AD. In humans, AD-causing mutations in APP and PS elevate plasma Aβ42 levels by 30%–100%, and are associated with the onset of dementia in the 3rd to 5th decade of life [6]. Studies of these same mutations in transgenic mice also demonstrate that small increases in Aβ42 levels markedly accelerate Aβ deposition in the brain and associated pathologies [7,8]. More recent studies in transgenic mice and Drosophila selectively expressing Aβ40 and Aβ42 in the secretory pathway, demonstrates that Aβ42 but not Aβ40 is sufficient to drive Aβ deposition, and, at least in Drosophila, neurodegeneration [9,10].

Although numerous lines of evidence support a role for Aβ42 in the underlying pathogenesis of AD, no therapies in clinical use target this molecule. The only currently approved treatments for AD are the acetylcholinesterase inhibitors (donepizil, rivastigmine, galantamine) and the N-methyl-D-aspartate receptor antagonist, memantine [11]. These pharmacologic therapies are thought to improve cognition by targeting specific symptoms of the disease, such as reduced cholinergic neurotransmission and increased glutamatergic activity leading to excess activation of NMDA receptors, respectively, without significantly modifying the underlying disease pathology [12,13]. Despite only modest symptomatic benefit, cholinesterase inhibitors have been widely adopted for clinical use in the early stages of AD, whereas memantine has shown benefit in the moderate to severe stages of the disease [14,15]. Given the growing AD epidemic there is an urgent need to discover alternative, more effective, therapies that not only target the disease symptoms but can also slow or halt the underlying neurodegenerative process. Several novel therapies based on the current understanding of AD pathogenesis are being clinically evaluated, including the use of anti-inflammatories [1].

Over the past 20 years, a substantial number of epidemiological reports have shown that long-term use of non-aspirin, nonsteroidal, anti-inflammatory drugs (NSAIDs) is associated with protection from the development of AD [16]. This data has been used to support the hypothesis that anti-inflammatory drugs may be effective in slowing the progression of disease, since a robust CNS inflammatory response is another prevalent feature of AD [17]. Indeed, the epidemiologic data has been used as the primary rationale for previous and ongoing trials of select NSAIDs in AD [18].

Results from these trials have been mixed. Clinical data from one double-blind placebo-controlled trial showed that indomethacin may improve cognition in dementia [19], but the results from this study were confounded by the lack of power and high drop-out rate due to adverse effects of the drug. A prospective, 25-week, randomized, double-blind placebo-controlled trial evaluating the efficacy and safety of diclofenac in combination with misoprostol in patients with mild to moderate AD in a prospective, 25-week trial, did not demonstrate a significant effect of NSAID treatment [20]. A more recent randomized, placebo-controlled 1-year clinical trial of naproxen or rofecoxib failed to detect any effect on cognitive impairment by naproxen or rofecoxib administration in mild to moderate AD patients [21]. Finally, because of concerns over side-effects, a large prevention study (the ADAPT trial) of naproxen and celecoxib was recently halted [22,23].

There are a number of possible reasons for the varying results from these trials, and one potential explanation comes from studies which suggest that the general anti-inflammatory activity of NSAIDs may play a secondary role in modulating the development of Aβ pathology while other pathways are involved [18]. First, chronic administration of only certain NSAIDs in mice has been reported to reduce Aβ deposition. Ibuprofen treatment was shown to significantly reduce amyloid pathology, neuritic dystrophy, plaque-associated gliosis and IL-1 expression in Tg2576 transgenic mice [24]. After 6 months of treatment, amyloid plaque numbers and Aβ levels in brain were reduced almost 50% and 40%, respectively. In the same study, naproxen treatment was not effective. This result suggests that cyclooxygenase (COX) inhibition, the main target of NSAIDs, *per se*, is not driving reductions in Aβ deposition. Furthermore, two nitric oxide-releasing derivates of flurbiprofen (HCT 1026 and NCX2216) and indomethacin have also been shown to reduce amyloid pathology following long-term administration to Aβ-depositing mice [25-27].

In contrast, nimesulide and celecoxib have been reported to have no effect on Aβ plaque pathology, further suggesting that COX 1 or 2 inhibition is not sufficient to decrease Aβ deposition [27,28]. These results provide additional evidence that only certain NSAIDs are able to suppress Aβ aggregation in the brain and that these compounds may be targeting a unique pathway.

One possible explanation for these observations is the discovery that certain NSAIDs, such as ibuprofen, indomethacin, and the enantiomer of flurbiprofen are capable of lowering the production of Aβ42 selectively [29,30]. In contrast, certain COX-2 inhibitors selectively raise Aβ42 or have no effect [31]. The ability of these compounds to modulate Aβ42 levels is independent of COX and other previously identified targets of these drugs, such as NFκB [32]. Although the mechanism of Aβ modulation has not been definitively established, experimental evidence suggests that these compounds target the γ-secretase complex which is composed of PS1 or PS2, substrate (i.e. carboxyl-terminal fragments of APP), and three essential accessory proteins: nicastrin, APH-1, and PEN-2 [30,33-35].

The identification of compounds that selectively lower Aβ42 provides a unique opportunity to test the hypothesis that decreasing levels of this peptide will have a positive impact on Aβ plaque pathology and cognition. Unfortunately, the potential toxicity related to inhibition of COX as well as diverse secondary targets of this class of drugs, complicates efforts to experimentally test this hypothesis. Extensive screening of NSAIDs, derivatives and related compounds led to the identification of *R*-flurbiprofen as a promising selective Aβ42-lowering agent that may circumvent some of these complications [30].

*R*-Flurbiprofen is a purified enantiomer of the classical racemic NSAID, flurbiprofen, which displays minimal COX activity and does not undergo stereoinversion in humans [36,37]. Like the racemate, *R*-flurbiprofen retains the ability to lower Aβ42 in cell culture and in the brain of young non-depositing Tg2576 APP mice following 3-days of oral dosing [30,38]. Based on *R*-flurbiprofen's selective lowering of Aβ42, reduced COX activity and safety profile in humans we have previously suggested that this drug was a good candidate for clinical testing in AD [30].

Recently, a 1 year randomized, placebo-controlled, double-blind Phase 2 study of *R*-flurbiprofen (MPC-7869, Myriad Pharmaceuticals, Inc.) in 207 subjects with mild to moderate AD (MMSE 15–26) was completed [39]. In mild AD subjects (MMSE of 20–26) receiving the highest dose of *R*-flurbiprofen (800 mg twice daily), statistically significant benefit was observed in measures of activities of daily living (ADCS-ADL; and global function (CDR-sb), with positive trends in cognition (ADAS-cog). No benefit was observed in moderate AD patients. In addition, *R*-flurbiprofen was generally well-tolerated in this patient population. Phase 3 studies are currently ongoing to further assess efficacy, safety, and the potential for this compound to be a disease modifying agent.

In this study we report the effect of chronic *R*-flurbiprofen treatment on cognition and Aβ loads in a transgenic model of AD, Tg2576 APP mice. Tg2576 mice were generated as follows: male Tg2576 (C57BL/6.SJL, APP+/-) were crossed with C57BL/6.SJL F1 females (APP-/-). These crosses generated the F2 Tg2576 APP +/- mice (mixed C57BL/6.SJL background), which were used in all experiments. In two separate behavioural trials, long-term treatment initiated in young Tg2576 mice with 10 mg/kg/day *R*-flurbiprofen improved spatial learning as assessed by the Morris water maze (WM). A modest reduction in biochemical Aβ loads was also observed, though this did not reach statistical significance in either study. A 2-week treatment of older Tg2576 with the same dose of *R*-flurbiprofen decreased Aβ plaque levels (p < 0.0001) but did not result in any significant alteration in spatial learning.

## Results

### Chronic administration and brain levels of *R*-flurbiprofen in Tg2576 mice

Acute 3-day oral dosing of *R*-flurbiprofen in Tg2576 mice is well-tolerated at doses up to 50 mg/kg/day. Maximal brain Aβ42 lowering was observed with doses of 25–50 mg/kg/day, but doses of 10 mg/kg/day also lowered Aβ42 [30]. In these initial short-term dosing studies no mortality or morbidity was observed. However, initial pilot long-term studies using dosing regimens designed to deliver either 25 and 50 mg/kg day (see Methods) resulted in 85% and 100% mortality, respectively, within 2 weeks. A 10 mg/kg/day dose did not result in increased mortality after 14 days, and was therefore chosen for long-term studies. Experimental design, group sizes, and survival data for each of the experiments designed to deliver ~10 mg/kg/day of *R*-flurbiprofen are shown in Tables 1 and 2. *R*- and *S*-flurbiprofen levels in the brain of mice from one water maze (Exp. 3) were analyzed using liquid chromatography and mass spectrometry techniques and were found to be 158 ± 14.7 and 76.0 ± 8.0 ng/gm brain tissue respectively.

**Table 1:** Experimental Design

**Experiment**	**Drug Regimen**	**Age at Drug Onset**	**Age at Behavioral Testing**	**Age at Sacrifice**
Experiment 1	Preventative	8 months	11½–12 months	15 months
Experiment 2	Preventative	8–9 months	11½–12 months	13 months
Experiment 3	Therapeutic	17 ½–18 months	18–19 months	19–20 months

**Table 2:** Subject

Water Maze	**Control Groups** Tg2575 mice not administered *R*-fluribiprofen				**Drug Treatment Groups** Tg2576 mice administered 10 mg/kg *R*-flurbiprofen			
	Initial N	**Final N**	Died	Failed Cue Training or Blind	Initial N	**Final N**	Died	Failed Cue Training or Blind

**Exp. 1**	18	**13**	3	2	13	**11**	0	2
**Exp. 2**	17	**13**	1	3	14	**10**	1	3
**Exp. 3**	15	**12**	0	3	15	**13**	1	1

### Sensory and motor ability

**Figure 1:**
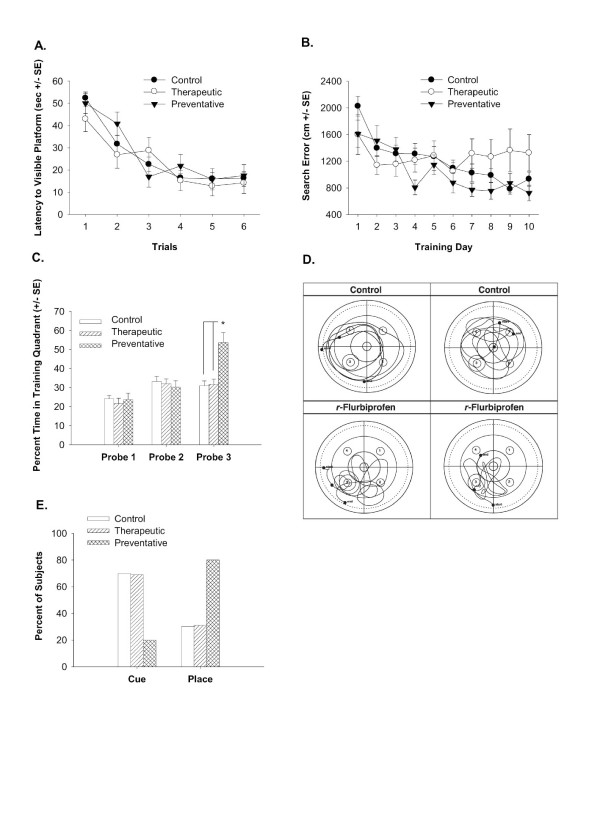
**Sensory motor and spatial learning ability measured in the water maze**. 1A shows the latency to escape to the visible escape platform during cue training. There are no differences in the performance of any of the groups. 1B shows the search error during the acquisition of the spatial reference memory task. The 2-week *R*-flurbiprofen-treated mice (Therapeutic group) performed more poorly than the other groups (Preventative group) on days 4, 7, 8, 9 and 10. 1C shows that only the Tg2576 mice treated for 16 weeks (Preventative group) with *R*-flurbiprofen developed a spatial bias for the training quadrant. 1D shows representative swim paths of the Control and the Preventative groups during Probe Trial 3. Note that the *R*-flurbiprofen-treated mice focus their search in the training quadrant that contained the hidden escape platform (bottom left quadrant). 1E shows the percent of subjects that preferred using a cue or place strategy during the strategy competition. 80% of the Tg2576 mice in the Preventative group preferred using a place strategy compared to only 30% of the Control and 31% of the Therapeutic group (Chi-square = 11.23, p < .01).

For the behavioural analysis, the control groups were collapsed across experiments as described in the statistical analysis section, resulting in 3 groups for statistical analysis: Control, Preventative ("Preventative", experiments 1 & 2) and Therapeutic ("Therapeutic", experiment 3)(see Statistical Analysis section for further discussion of groups). Assessment of sensory and motor ability was determined by measuring the latency of mice to escape to a visible platform and their swimming speed. Figure 1A shows that there were no differences between groups in escaping to the visible platform [F(2,55) = .61, ns] although all groups became more proficient in escaping to the visible platform over the course of the 6 training trials [F(55,5) = 31.62, p < .0001]. Similar results were apparent in the pathlength the mice took to reach the visible escape platform, which showed no main effect of group [F(2,55) = 1.23, ns] but a general decrease in distance travelled with increased training [F(55,5) = 28.35, p < .0001, data not shown]. Table 3 shows that mice treated with *R*-flurbiprofen, regardless of length of administration, had modest but significantly lower swimming speed [F(2,55) = 4.11, p < .05].

**Table 3:** Swim Speed

**Group**	**Swim Speed **(m/sec ± SE)
Controls	.191 ± .005
Preventative (Exp. 1 & 2)	.169 ± .010*
Therapeutic (Exp. 3)	.167 ± .008*

*p < .05 compared to Controls, Fisher's PLSD

### Spatial reference memory

Figure 1B shows the search error in the acquisition of the spatial reference memory task. All groups became more proficient with increased training [F(55, 9) = 8.38, p < .0001]. A significant interaction between training day and group indicated that the groups differed on certain days but there was no main effect of drug-treatment group [F(2,55) = 1.16, ns]. *Post hoc *Fisher's PLSDs for each day revealed the following differences: on day 4, there was a significant difference between the Control and the Preventative group (p < .05); on days 7, 8, and 10 there was a significant difference between the Therapeutic and the Preventative *R*-flurbiprofen groups (p < .05); on day 9 there was a significant difference between the Control and Therapeutic group (p < .05). The general pattern indicates that the oldest group of mice that only received drug for 2 weeks (Therapeutic) performed more poorly than the vehicle and 16-week *R*-flurbiprofen-treated (Preventative) groups on the last few days of training trials. Similar results were obtained with latency (day × group interaction, F(2,55) = 1.77, p < .05) and pathlength measures (day × group interaction, F(2,55) = 2.02, p < .01), data not shown.

The spatial bias of the mice for the location of the hidden escape platform is shown by the percent of time the mice spent in the quadrant of the maze that contained the platform during the three interpolated probe trials (Figure 1C). A main effect of group [F(2,55) = 3.91, p < .05] and a significant interaction between group and probe trial [F(4,110)= 6.52, p < .0001] indicates that the mice in the Preventative *R*-flurbiprofen treatment group significantly increased their spatial bias on the third probe trial (post hoc one-way ANOVA (× Group) on Probe 3, [F(2,55) = 12.10, p < .0001]). The performance of the Preventative group is significantly different from both the Control and the Therapeutic group (Fisher's PLSD p's < .0005). Figure 1D shows representative swim paths of the control and the Preventative group during the third probe trial. The swimming paths of the *R*-flurbiprofen-treated mice demonstrate a more localized search pattern over the location of the hidden escape platform (bottom left quadrant), indicating a well-formed spatial bias.

### Strategy preference

In accordance with the increased spatial bias of the mice on the *R*-flurbiprofen Preventive regimen, these mice also show a significant preference for using a hippocampal-dependent place learning strategy compared to the Control and Therapeutic groups (*χ*^2 ^= 11.23, p < .01), shown in Figure 1E. The Control and the Therapeutic groups both preferred the use of a cue response on the competition test, thought to indicate the use of a striatal-based learning strategy [40].

### Assessment of Aβ loads

At the conclusion of behavioural testing, mice were sacrificed and assessed for biochemical loads of Aβ using sequential extraction first of detergent soluble Aβ followed by formic acid (FA) soluble Aβ. Although both groups of mice on the preventive dose of *R*-flurbiprofen (experiments 1 & 2) displayed similar improvements of spatial learning and were pooled for analysis of behaviour, they were aged for different lengths of time following the end of water maze testing and were expected to have different levels of Aβ deposition (see Table 1). Thus, these groups were separated for biochemical analysis of Aβ levels.

**Figure 2:**
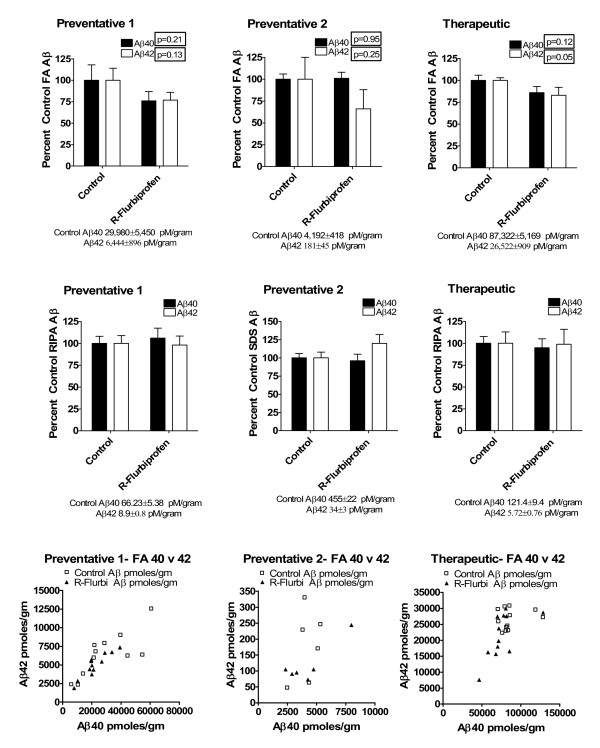
**ELISA quantitation of Aβ levels in Tg2576 from water maze experiments**. The levels of detergent soluble (RIPA or SDS) and formic acid soluble Aβ1–40 and Aβ1–42 in *R*-flurbiprofen treated mice from the three water mazes are plotted as percent control. The mean Aβ levels +/- the standard error from control mice are shown below each graph. The absolute values (pmoles/gram tissue) of formic acid soluble Aβ40 and Aβ42 levels from individual animals are plotted against one another to show the distribution of individual animals from experimental groups. "Preventative 1 and Preventative 2" refers to two different experiments examining the effects of 4 months of administration of *R*-flurbiprofen to Tg2576 mice. "Therapeutic" refers to 2 weeks administration of *R*-flurbiprofen.

**Figure 3:**
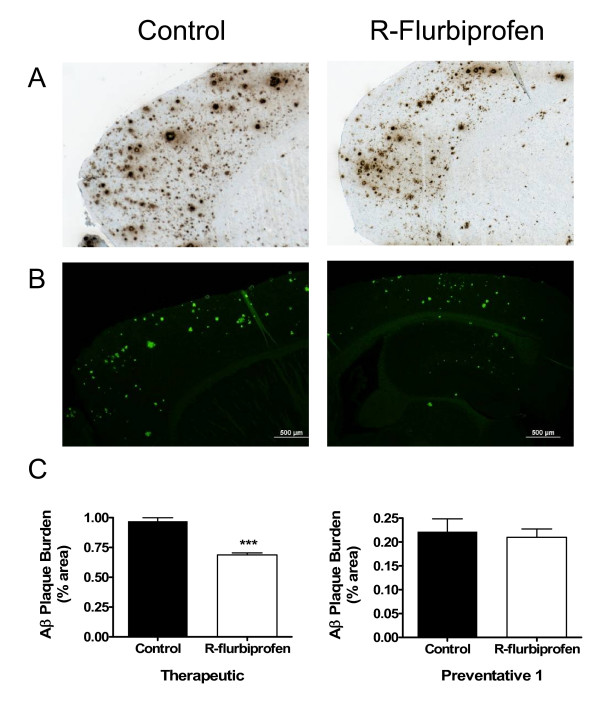
**Effect of *R*-flurbiprofen treatment on amyloid-β plaques in Tg2576 mice**. Brain sections of representative Control and R-flurbiprofen-treated mice from water maze Experiment 3 (20 months at sacrifice) were stained for Aβ plaques using antibody 33.1.1 (A) and thioflavin (B). In (C), the level of Aβ plaque burden (quantified as % Aβ immunoreactive area) in Control compared to R-flurbiprofen treated-Tg2576 mice from Experiments 2 and 3 are compared. Note the significant reduction in plaque burden in the R-flurbiprofen treated mice from Experiment 3 (*** p < 0.0001).

We observed no significant differences in detergent soluble Aβ40 or Aβ42 levels between the treated and control groups in experiments 1, 2 or 3 (Fig 2) as measured by peptide specific sandwich ELISAs. In experiment 1, preventative *R*-flurbiprofen treatment and sacrifice at 15 months of age resulted in a non-significant reduction in FA Aβ40 (24%) and Aβ42 (23%) levels (Fig 2). In experiment 2, a preventative *R*-flurbiprofen regime and sacrifice at 13 months of age produced no change in Aβ40 levels but a 34% decrease in the levels of FA Aβ42, which did not reach significance (p < 0.25). In experiment 3, a 2-week therapeutic treatment with *R*-flurbiprofen in 20-month-old mice produced a non-significant lowering of Aβ40 (14%) and a significant lowering of Aβ42 (17%) (p < 0.05). Assessment of total Aβ plaque load using immunohistochemical quantification failed to reveal significant differences after long-term *R*-flurbiprofen treatment (experiment 2). In contrast, short-term treatment with *R*-flurbiprofen at 20 months of age (experiment 3) resulted in a significant decrease in Aβ plaque burden as measured by the percent immunoreactive area (Fig 3C, p < 0.001).

## Discussion and conclusion

We have found that chronic administration of *R*-flurbiprofen is able to attenuate spatial learning deficits in Tg2576 mice that began treatment at 8 months of age. The improved spatial learning ability of the *R*-flurbiprofen-treated mice was indicated by an increased spatial bias during the third probe trial and the increased preference for using a place strategy on the strategy preference test. These results are consistent with an improvement in hippocampal/medial temporal lobe-dependent memory function [40]. A short 2-week administration of *R*-flurbiprofen in 19-month-old Tg2576 mice with AD-like Aβ loads had no effect on cognitive performance. Unfortunately, long-term treatment of aged Tg2576 mice with the same dose of *R*-flurbiprofen results in enhanced morbidity and mortality (not shown), making it difficult to perform an experiment in which older mice are treated for the same period of time.

Both long- and short-term treatment of Tg2576 mice with *R*-flurbiprofen resulted in a decrease in the swim speed of the mice. It is possible that the decrease in swim speed in the *R*-flurbiprofen-treated mice observed in this study reflects a general effect of returning motor activity to normal levels in Tg2576 mice. Decreases in swim speed reported in this study are unlikely to account for the effects of *R*-flurbiprofen on our measures of hippocampal-dependent memory as *R*-flurbiprofen was an effective modulator of swim speed with both the Preventative and Therapeutic treatment regimens but only the Preventative treatment regimen increased spatial learning ability in the Tg2576 mice.

*R*-flurbiprofen has been demonstrated to lower steady-state Aβ42 levels after 3 days of oral administration in Tg2576 mice [30]. In this study, however, the results indicate that a preventative treatment strategy of *R*-flurbiprofen administration does not significantly alter soluble, RIPA extracted-Aβ40 or 42 but does reduce insoluble, formic acid-soluble Aβ levels 24–34%, although the decrease is not statistically significant (Fig 2). Interestingly, the preventative treatment had no effect on plaque burden (Fig 3). These results contrast with the therapeutic, or short-term, treatment in 18-month-old mice where formic-acid soluble Aβ42 levels and the Aβ plaque burden was significantly decreased. The reduction in Aβ42 levels and plaque burden did not improve spatial learning in the 18-month-old mice. Clearly, these results indicate that the length of time *R*-flurbiprofen is administered and the age at which treatment is started are critical factors in producing beneficial effects on cognition. The relationship between cognitive improvement and Aβ is controversial. Improvement in spatial memory in Tg2576 mice has been shown to occur using a preventative regime of ginko biloba or alpha lipoic acid without a corresponding change in Aβ levels or deposition [41,42]. In addition, several studies now show that cognitive impairment in Tg2576 mice and other APP mice does not correlate well with Aβ deposition and may be attributable to small soluble assemblies of Aβ [43,44]. Moreover, cognitive improvement can be seen following anti-Aβ immunotherapy, even when there is no significant alteration in Aβ deposition [45-49]. It is possible that delaying interventions until pathology is well-developed may prevent functionally significant changes in learning and memory due to irreversible changes in synaptic transmission.

The reason that the two-week therapeutic-regimen in older Tg2576 (19–20 month) did not lead to significant behavioral improvement is not readily apparent. However, since it appears that chronic treatment with *R*-flurbiprofen is necessary in Tg2576 mice for spatial learning augmentation, it seems unlikely that the drug is working through the same mechanism as the anti-Aβ antibodies, which have been reported to work rapidly [48]. Furthermore, our more recent experience with behavioral testing of APP mice suggests that negative results be interpreted cautiously, since assessment of the cognitive phenotype in APP mice can be variable even among cohorts of mice bred and housed in the same environment (unpublished observations). Moreover, it is possible that behavioral testing and analysis of Tg2576 mice itself alters Aβ burden since it has been shown that enrichment paradigms, such as access to running wheels, appear to be capable of altering Aβ deposition and cognition [50,51]. In future studies it will be important to control for possible effects of behavioral training on Aβ deposition by including cohorts of treated and untreated mice that are not subject to behavioral testing.

In humans, *R*-flurbiprofen appears to be safe and is well-tolerated, with relatively few side effects [39] and undergoes minimal racemization to the *S*-enantiomer [36]. In contrast, following oral administration to mice ~20–40% of the *R*-flurbiprofen is converted to the S-enantiomer (see Results; [52]). This conversion is likely to account for toxicity observed upon chronic dosing regimens more likely to alter Aβ42 production. Overall, our data demonstrate a clear cognitive benefit of long-term administration, but are inconclusive with respect to the relationship between effects on Aβ deposition and behavioural improvements. Previous studies have shown that chronic treatment with nitric oxide-releasing flurbiprofen derivatives do lower Aβ accumulation in APP mice, but it is not clear if this effect is due to the prodrug (which is not likely to affect Aβ42 production) or flurbiprofen released following metabolism [27,53]. In this study we see trends towards reduced Aβ deposition in the prevention studies and small but significant decrease in Aβ deposition in the therapeutic study.

Aβ42-lowering NSAIDs have been shown to affect multiple pathways and targets that could alter the AD phenotype, any of which could contribute to their ability to protect against the development of AD [18]. For instance, certain NSAIDs reduce production of the more amyloidogenic Aβ peptide by altering γ-secretase cleavage of APP [29,30]. While other NSAIDs have also been shown to directly inhibit Aβ aggregation *in vitro *[54,55]. They may also decrease BACE expression through activation of PPARγ leading to decreased levels of Aβ [56]. In addition, more classic anti-inflammatory activities against cyclooxygenase as well as additional non-intended target activities against lipoxygenase and NFk-β could mediate beneficial effects [18]. In contrast, *R*-flurbiprofen is not a classic NSAID in humans because it does not significantly inhibit cycloxygenase enzymes; however, it is similar in structure and could bind to other known targets of NSAIDs as well as unknown targets. Given the data presented here which suggests that *R*-flurbiprofen improves cognitive performance in APP mice it will be important in future studies to further delineate the underling mechanisms that contribute to efficacy of *R*-flurbiprofen as a potential AD therapeutic.

Taken together, the results from this study show that chronic treatment with *R*-flurbiprofen beginning at an early stage of cognitive dysfunction maybe necessary to improve learning impairments, at least in the Tg2576 APP mouse model. An important caveat is that these results were obtained using a sub-optimal dose of *R*-flurbiprofen, which was necessitated by the toxicity of the compound at higher doses in these mice, most likely caused by bioinversion to *S*-flurbiprofen and the subsequent COX-mediated gastrointestinal toxicity that is not seen in humans. Thus, extrapolating this dosing paradigm and results to human AD patients is difficult at best. The ongoing phase 3 clinical trial of *R*-flurbiprofen in mild AD patients should provide the most conclusive test to date of the efficacy of this drug, and the general strategy of selectively lowering Aβ42, in modifying the underlying disease progression and associated cognitive decline in AD.

## Methods

### Animals

All animal husbandry and testing procedures performed were approved by the Mayo Clinic Institutional Animal Care and Use Committee in accordance with NIH guidelines. Female Tg2576 mice were generated and confirmed by genotyping as described previously [57,58]. All animals were housed 3–5 to a cage and maintained on ad libitum food and water with a 12-hour light/dark cycle in a controlled environment. One week prior to and during water maze testing animals were individually housed. *R*-Flurbiprofen, at a concentration of 67, 167.5 or 335 or mg per kg of diet was homogenously incorporated into Harlan Teklad 7012 kibble by Research Diets, Inc. Based upon dietary consumption of diet at this age, these diets were designed to result in 10, 25 or 50 mg *R*-flurbiprofen on average per day. By monitoring daily consumption on the dose designed to deliver 10 mg/kg/day, we estimate that the mice received between 8–12 mg *R*-flurbiprofen per kg body weight per day. Kibble consumption, general health and body weight were monitored on a weekly basis. For behavioral experiments, all mice were negative for the retinal degeneration mutation that occurs in approximately 25% of the mice in our colony [59].

### *R*-Flurbiprofen experiments

The mice were assigned to a "Preventative" or a "Therapeutic" experimental group based on age. *R*-Flurbiprofen was administered at a concentration of 10 mg/kg/day for all experiments. In the two "Preventative" experiments (experiments 1 and 2), dosing was started at ages prior to or early in the emergence of cognitive deficits, between 8 and 9 months of age, and treatment continued for 4 months. Treated and untreated mice were then assessed for spatial learning ability in the Morris water maze at approximately 12 months of age when learning deficits are usually present (e.g. [43]). In a "Therapeutic" experiment (experiment 3), Tg2576 mice were given 2 weeks of *R*-flurbiprofen prior to water maze testing, starting at 18 months of age when Aβ levels have already increased and cognitive deficits are normally present.

### Spatial learning assessment

At the conclusion of the drug-treatment phase, mice were assessed for spatial reference memory in the Morris water maze. Subjects were maintained on their appropriate drug regime during behavioral evaluation. The water maze was chosen as the testing paradigm as spatial learning in the water maze has been extensively used to measure hippocampal function and age-associated cognitive impairment in rodents (e.g., [60]).

The water maze consists of a circular tank (4-ft dia) with a removable escape platform centered in one of the four maze quadrants. During testing, the tank is filled with 25°C water clouded by non-toxic white paint. For training trials, the top of the escape platform is submerged .05 cm below the water surface. Black curtains with white patterns surround the maze. Data are analyzed using a video tracking system and software developed by Richard Baker, HVS Image Analyzing (Hampton, U.K.).

Cue training occurs prior to hidden platform trials to test for sensorimotor and motivational factors that may influence spatial learning. There is one day of cue training, containing 6 trials. A visible black platform that extends 2 cm from the water surface is moved to different locations in the pool between trials and the subject's entry point into the tank randomized. Each mouse is given 30 sec to reach the platform and remains on the platform briefly. Trials are separated by a 5–10 minute intertrial interval. Mice that performed +/- three times the standard deviation from the mean were excluded from further study. This accounted for approximately 20% of the initial number of mice. In addition, mice that exhibited stereotypic behaviour such as compulsive circling were also excluded from behavioral analysis (< 1%).

For hidden platform training trials, there are 4 trials a day for 10 days. The animal is placed in the water at the pool's perimeter and allowed 60 seconds to locate the stationary escape platform. These trials generate the search error score that reflects the animal's distance from the platform throughout their search. The search error reflects the cumulative proximity of the mouse from the escape platform during the training trial. The position of entry for the animal is varied at each trial. There is a 5–10 minute inter-trial interval. The first trial of training days 4, 7, and 10 consists of a 30 sec probe trial that serves to assess the development of a spatially localized search for the escape platform. During such trials, the escape platform is unavailable for escape.

Strategy preference was assessed 24 hours after the last day of place training. A visible platform was placed in the quadrant opposite the training quadrant where the hidden platform was previously located. The mice were given two 60-second strategy probe trials. Start locations were on either side of the tank, equidistant from the visible cue platform and the prior hidden platform location. A "place strategy" was recorded if the mouse crossed the annulus of the prior hidden platform location before escaping to the visible platform. The annulus was defined as a 5 cm perimeter around the prior hidden escape platform location. A "cue strategy" was recorded if the mouse did not cross the prior hidden platform annulus before swimming to the visible platform. Data was scored by observation of recorded swim paths by the HVS Imaging program.

### Analysis of flurbiprofen levels in brains of Tg2576 mice

Mouse hemibrains or cerebella were homogenized in two volumes of HPLC-grade water (μL per mg) with a Turrax T8 homogenizer followed by centrifugation of homogenates at 2,000 g 4°C, for 10 min. Concentrations of *R *and *S*-flurbiprofen in extracts were determined against untreated brain extracts including drug standards, with a range of quantification between 1–1,000 ng/ml using tandem liquid chromatography-mass spectrometry-mass spectrometry as previously described [31].

Methanol (200 μL) was used to precipitate proteins from 100 μL of brain homogenate followed by fortification with an internal standard (deuterated racemic flurbiprofen). Samples were mixed for two minutes in a Captiva filter plate on a plate shaker before being transferred to a vacuum apparatus. Vacuum (1 mm Hg) was applied for three minutes and filtered extracts were collected in a new 96-well plate ready for LC-MS/MS analysis.

A calibration curve for the quantitative determination of each compound was made in the range of 1 to1000 ng/mL. Each 10× spiking solution (10 μL) was added to 90 μL of blank mouse brain homogenate (prepared as above) to generate the curve. Three QC samples were made at each different concentration of 80, 320 and 800 ng/mL to determine the validity of the calibration curve. Standard curve and QC points were prepared in the same manner as the samples for analysis.

Following sample preparation, 10 μL of sample extract was injected onto a Daicel ChiralPAK AD-RH 4 × 150 mm column and eluted at 0.55 mL/min using the following isocratic mobile phase: 90% methanol, 5% acetonitrile, 5% water and 0.1% acetic acid. Compounds were detected using an ABI 4000 Q-Trap linear ion trap mass spectrometer in Multiple Reaction Monitoring mode with the following mass transitions monitored: m/z 243.3→199.1 for 7869 and *S*-flurbiprofen and m/z 246.3→202.1 for the deuterated 7869 and *S*-flurbiprofen internal standard.

### Analysis of Aβ levels in Tg2576 mice

Brains of mice were divided by midsagittal dissection after sacrifice by CO_2 _asphyxiation. One hemibrain was fixed in 4% paraformaldehyde for immunohistochemical studies and the other flash frozen in isopentane and used for biochemical analysis as described previously [61]. Briefly, each hemibrain (150 mg/ml wet wt) was extracted in either 1× RIPA buffer or 2% SDS with complete protease inhibitor (Roche) using a PowerMax 200 homogenizer (VWR), followed by centrifugation at 100,000 g for 1 hour at 4°C. Following centrifugation, the supernatant was collected, which represented the detergent-soluble fraction. The resultant pellet was then extracted in 70% FA, using a probe sonicator 3000 (Misonix), centrifuged at 100,000 g for 1 hour at 4°C, and the supernatant collected. Extracted Aβ was measured using a sandwich ELISA system utilizing monoclonal antibodies developed at Mayo Clinic that has been described before in detail [31,61]. To measure Aβ42 levels-peptides were captured with mAb 2.1.3 (specific for the c-terminus of Aβ42) and detected with HRP-conjugated mAb Ab9 (specific for human Aβ1–16); To measure Aβ40 levels peptides were captured with mAb Ab9 and detected with HRP-conjugated mAb 13.1.1 (specific for the c-terminus of Aβ40)

### Immunohistology

Hemibrains of mice were fixed in 4% paraformaldehyde in 0.1 M PBS (pH 7.6) and then stained for Aβ plaques as described previously [61]. Paraffin sections (5 μm) were pretreated with 80% FA for 5 minutes, boiled in water using a rice steam cooker, washed, and immersed in 0.3% H2O2 for 30 minutes to block intrinsic peroxidase activity. They were then incubated with 2% normal goat serum in PBS for 1 hour, with 33.1.1 (Aβ1–16 mAb) at 1 μg/ml dilution overnight, and then with HRP-conjugated goat anti-mouse secondary mAb (1:500 dilution; Amersham Biosciences) for 1 hour. Sections were washed in PBS, and immunoreactivity was visualized by 3,3'-diaminobenzidine tetrahydrochloride (DAB) according to the manufacturer's specifications (ABC system; Vector Laboratories). Adjacent sections were stained with 4% thioflavin-S for 10 minutes. Fixed, paraffin-embedded sections were stained for activated microglia using anti-Iba1 (1:3000; Wako Chemicals) and for activated astrocytes using anti-GFAP (1:1000, Chemicon).

### Quantitation of amyloid plaque burden

Computer-assisted quantification of Aβ plaques was performed in a blinded fashion as described previously [62]. Serial coronal sections were stained with 33.1.1 and plaques were quantified (calculated as proportional area of plaque burden) in the neocortex of the same plane of section for each mouse (~10 sections per mouse) using MetaMorph 6.1 software (Universal Imaging Corp, Downington, PA).

### Statistical analysis

Experiments 1 and 2 were replicate experiments investigating the long-term treatment effects of *R*-flurbiprofen on spatial memory. The results from the two studies paralleled each other, but the N was relatively small in experiment 2 compared to experiment 1 (experiment 1, control N = 12, *R*-flurbiprofen = 9; experiment 2, control = 6, *R*-flurbiprofen = 6). There were no differences between the 12-month-old control mice from experiments 1 and 2 (N = 18) when compared to the 19-month-old control mice from experiment 3 (N = 12) on visible platform latency [F(1,28) = .15, ns], hidden platform search error [F(1,28) = .003, ns] or the percent time in training quadrant [F(1,28) = .05, ns]. Therefore, for subsequent analysis of the behavioral data, the control groups from all three experiments were collapsed into one group for statistical power and clarity of presentation. The final groups were: vehicle control (N = 30), the collapsed group of experiments 1 and 2 (16-week-*R*-flurbiprofen treatment, preventative regimen, N = 15) and experiment 3 (2-week-*R*-flurbiprofen treated, therapeutic regimen, N = 15). Repeated measures ANOVAs (× Group) were then used to examine visible platform latency, hidden platform search error and probe trial data. Significant interactions were examined *post hoc *by one-way ANOVAs and Fisher's PLSDs. Chi-square analysis was done on the number of subjects categorized as "place" or "cue" learners during the strategy preference test. For presentation purposes, this data was converted to the percent of subjects.

## Abbreviations

Alzheimer's disease (AD); Alzheimer's Disease Cooperative Study Activities of Daily Living Index (ADCS-ADL); Alzheimer's Disease Assessment Scale cognitive subscale (ADAS-cog); amyloid beta peptide (Aβ); Amyloid Precursor Protein (APP); Clinical Dementia Rating-Sum of Boxes (CDR-SB); cyclooxygenase (COX); diaminobenzidine (DAB); enyzme-linked immunosorbent assay (ELISA); horseradish peroxidise (HRP); phosphate-buffered saline (PBS); Mini-Mental Status Examination (MMSE); Morris water maze (WM); non-steroidal anti-inflammatory drugs (NSAIDs); RIPA (radioimmuno precipitation assay); sodium dodecyl sulfate (SDS)

## Authors' contributions

TK participated in watermaze studies, performed Aβ ELISA measurements and analysis, participated in the conception, design and execution of the study and helped to draft the manuscript. SP participated in watermaze studies. JLE participated in watermaze studies, performed Aβ ELISA measurements and participated in the conception and design of the study. VH participated in Aβ histology and quantification. MPM carried out Aβ immunoassays. EK participated in the conception of the study. TEG participated in the conception, design and coordination of the study and helped to draft the manuscript. MMN participated in the conception, design, and coordination of the study, performed statistical analysis and helped to draft the manuscript.
